# Experimental Study of the Relaxation Properties of Carbon Fiber Cloth

**DOI:** 10.3390/ma13163603

**Published:** 2020-08-14

**Authors:** Qiang Wang, Hua-Lin Song, Chun-Ling Lu, Wan-Xu Zhu, Jia-Zhu Huang

**Affiliations:** 1 College of Civil and Architecture Engineering, Guilin University of Technology, Guilin 541004, China; 2000032@glut.edu.cn (Q.W.); songhualin@glut.edu.cn (H.-L.S.); zhuwanxu@vip.163.com (W.-X.Z.); 2 Guangxi Key Laboratory of New Energy and Building Energy Saving, College of Civil Engineering and Architecture, Guilin University of Technology, Guilin 541004, China; 3 Collaborative Innovation Center for Exploration of Nonferrous Metal Deposits and Efficient Utilization of Resources, Guilin University of Technology, Guilin 541004, China; 4 Operation department, Guilin High-performance Concrete Materials Technology Limited Liability Company, Guilin 541004, China; hjz15078351070@163.com

**Keywords:** carbon fiber cloth, relaxation performance, experimental study, calculation model, prestress loss

## Abstract

In this study, a new method was proposed to study the relaxation properties of carbon fiber reinforced plastics (CFRP) fabric under axial tension. Under the condition of constant temperature and humidity, six groups of 168 h stress relaxation tests were conducted. Considering the influence of the prestress level, the size of CFRP cloth, and the surface coating of CFRP cloth on the relaxation performance, the measures to reduce the relaxation loss were proposed. The relaxation rate calculation model was established based on the test results of the authors and other scholars and was validated through comparisons with the test results. The results indicate that the relaxation rate of CFRP cloth was between 1.92% and 6.1%. When the prestress level was smaller than 0.3 fu, the relaxation rate of CFRP cloth decreased with the increase of prestress level. When the prestress level was greater than 0.3 fu, the relaxation rate increased with the increase of the prestress level. Under the same conditions, the relaxation rate of the CFRP specimens coated with glue was smaller than the uncoated samples by 3.21–6.28%. The calculation model could well estimate the relaxation rate of CFRP cloth.

## 1. Introduction

Over the past decade, the frequent occurrence of natural disasters has made structural reinforcement a particular concern to countries all over the world. Fiber Reinforced Polymer (FRP) is a composite material composed of load-bearing fibers and a resin matrix. It is light (approximately 0.2 times of the weight of steel). It also has a high tensile strength (1000–5000 MPa for ordinary grade CFRP sheets) and excellent corrosion resistance. The FPR materials can be widely used for structural reinforcement because of their superior performance [1,2,3,4]. FRP reinforcement methods are currently applied to four types of FRP materials: aramid fiber-reinforced composite materials (AFRP), basalt fiber-reinforced materials (BFRP), glass fiber-reinforced composite materials (GFRP), and broadly used carbon fiber-reinforced composite materials (CFRP). The CFRP cloth is anisotropic. It is manufactured in bundles of thousands of tiny fibers and woven onto a fabric roll. Researchers have conducted extensive research on the performance of FRP materials to reinforce concrete members [5,6,7,8]. Compared with the traditional external FRP reinforcement method, the prestressed FRP technology as an active reinforcement method can avoid the stress-lag effect of the FRP materials. Moreover, it takes full advantage of the high-strength characteristics of the FRP materials, enhances the working performance and ultimate strength of the reinforced member, and effectively suppresses the deformation and crack development in the component of interest [9,10,11,12]. One of the most prominent problems of the prestressed FRP is stress relaxation, which is attributed to the decrease of stress with time during a constant deformation. To address this well-known problem, the study of the relaxation performance of the prestressed FRP materials, analysis of the relaxation rules and the relaxation quantity, and proposition of measures to reduce the relaxation loss are inevitable. They have to be carried out before applying the prestressed FRP materials for the reinforcement of engineering structures. In recent years, research on the prestressed FRP materials has focused on FRP rebars, FRP plates, and FRP cloth [13]. Saadatmanesh [14] and Tannous [15] studied the relaxation properties of AFRP rebars and CFRP rebars and found that there is a logarithmic relationship between relaxation and time. Through a regression analysis of the test data, Gunnarsson [16] predicted the time-dependent stress relaxation rate of FRP rebars. According to the regression equation obtained, when the initial stress was 0.5 fu, the 50 years stress relaxation rate of BFRP rebars was predicted to be 11.2%. This is much larger than the 50 years relaxation rate of BFRP rebars mentioned in the literature. The large difference originates from not considering the potential slippage of the anchor. To eliminate the effect of anchor slippage, Shi et al. [17] developed a test device and investigated the relaxation rate of BFRP rebars when the initial stress was 0.4, 0.5, and 0.6 fu. The test results indicate that the stress relaxation coefficients at 1000 h were 4.2%, 5.3%, and 6.4%, respectively. Guo et al. [18] conducted 30 days prestress monitoring on prestressed carbon fiber lamella (CFL) and found that the stress loss rates of the test piece were 1.8%, 1.9%, and 2.5% when the prestress levels were 0.08, 0.15, and 0.22 fu, respectively. The stress loss of CFL in the stable stage is related to the magnitude of prestress. The larger is the prestress, the larger is the loss of the prestress in the stable stage. Huang et al. [19] monitored the pre-stress loss tests of a 20 day-old carbon fiber board and performed regression analysis on the test data. The tests indicated that the relaxation loss of the CFRP board increased with the increase of the initial stress and time. In addition, the relaxation loss developed rapidly in the early stage and became steady after 100 h.

Research attempts have been conducted on the loss of prestressed CFRP cloth in strengthened reinforced concrete (RC) members. Kim et al. [20] proposed a closed solution to the stress analysis of the bond interface for the short-term prestress loss of the post-tensioned CFRP cloth in the reinforced prestressed concrete beam. Through calculation, it was found that the prestress loss was about 10% of the initial prestress. Costa et al. [21] found through experimental observations that the effective stress of prestressed FRP materials is linearly linked to the ambient temperature. Hence, the change of the ambient temperature is the key parameter for the correct evaluation of the effective stress. Zhou et al. [22] reinforced five concrete columns and five steel columns with pre-stressed CFRP fabrics. The pre-stress loss mainly stems from CFRP fabric stress relaxation loss and friction loss. The relaxation loss of CFRP cloth consists of two main components: (1) the stress redistribution due to the mutually coordinated deformation between fibers; and (2) stress relaxation of the carbon fiber itself. Lu et al. [23,24] conducted loss tests on cylindrical and square columns of reinforced concrete reinforced by prestressed CFRP cloth and measured the relaxation loss. The test results suggest that closer to the end of the anchor root there is greater relaxation loss of the fiber cloth. In addition, the lack of friction at the distal end of the fiber cloth anchor root can cause a negative increase in relaxation loss over time. According to the above-mentioned literature reports on the study of stress loss, the method of strengthening the RC member and then analyzing the relaxation loss of the fiber cloth results in an inaccurate analysis of the relaxation loss of the CFRP cloth. This comes from large differences in the relaxation loss of the fiber cloth in the circumferential reinforcement positions, deformation of the anchor, and also deformation coordination between fibers.

Chinese scholars have investigated the relaxation properties of CFRP fabrics under axial tension. Wang et al. [25,26] proposed the calculation equation of the prestress loss using the relaxation test of the CFRP specimen. Li et al. [27,28] studied the stress relaxation behavior of four CFRP fabric specimens under axial tension. They analyzed the effect of the prestress level on relaxation loss and proposed the time-dependent relaxation rate equation of CFRP fabric. Deng et al. [29] analyzed the stress relaxation test results of aramid fiber fabric under axial tension and proposed a equation for calculating the relaxation rate that changes with time using regression. The above literature has few considerations for the relaxation rate of the CFRP fabric under axial tensile conditions. This significantly limits the application of the calculation results.

Based on the aforementioned literature review and the aforesaid research deficiency, to make full use of the potential of CFRP and to understand the relaxation characteristics of prestressed CFRP cloth, an independently developed device was employed in this research to conduct six groups of stress relaxation tests that eliminate the slippage caused by CFRP cloth. Considering the effect of prestress level, CFRP cloth ruler, and glue treatment on relaxation performance, some measures were proposed to improve the relaxation rate. The relaxation rate equation of CFRP cloth was established and compared with other research results. The equation provides a reference for the application of CFRP as prestressed reinforcement material and for the improvement of its utilization efficiency in construction.

## 2. Test Overview

### 2.1. Specimen Parameters and Material Mechanical Properties

**Figure 1:**
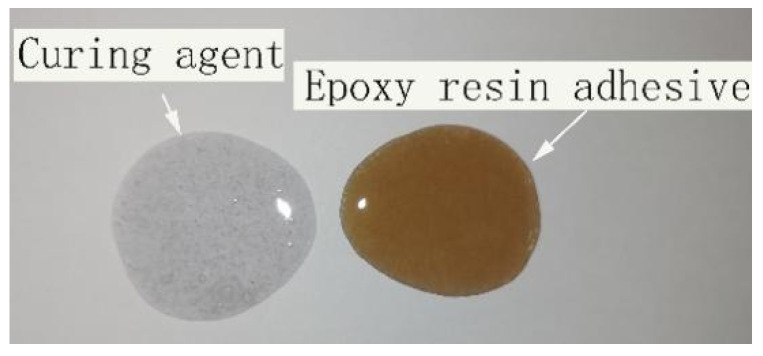
Dip adhesive.

**Figure 2:**
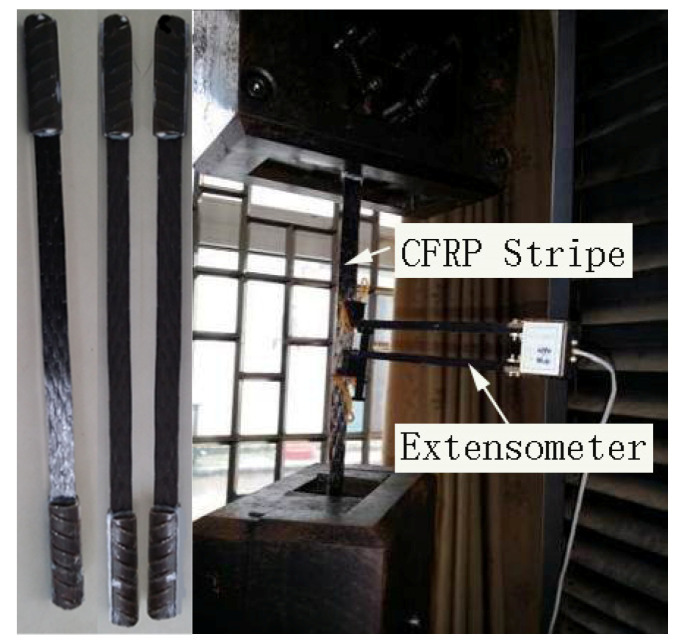
performance test of CFRP cloth.

To study the relaxation performance of CFRP, the test was conducted at a constant temperature and constant humidity environment with a temperature of 20 ± 2 °C and relative humidity of 55–60%. In this experiment, six groups of test pieces were designed, with a total of 24 pieces. The tensile lengths of the test pieces were 600, 1200, and 2400 mm, and the width of the cloth was 150 mm. The prestress levels of each group were 0.1, 0.2, 0.28, and 0.34 fu, respectively, and the last three groups of specimens were coated with glue. The parameters of the test pieces of each group are shown in Table 1. The CFRP used in the test and the impregnating adhesive matched with the CFRP are produced by Carbon Composites Co., Ltd., Tianjin, China. The CFRP model is CFS-I-300, and the single-layer thickness is 0.167 mm. The dip adhesive consists of two-component: epoxy resin adhesive and curing agent, which were mixed with a 2:1 ratio (see Figure 1). The performance test of the CFRP cloth that was conducted in the 200-t universal testing machine is shown in Figure 2. The mechanical properties of the CFRP cloth and the impregnated adhesive are also presented in Table 2.

**Figure 3:**
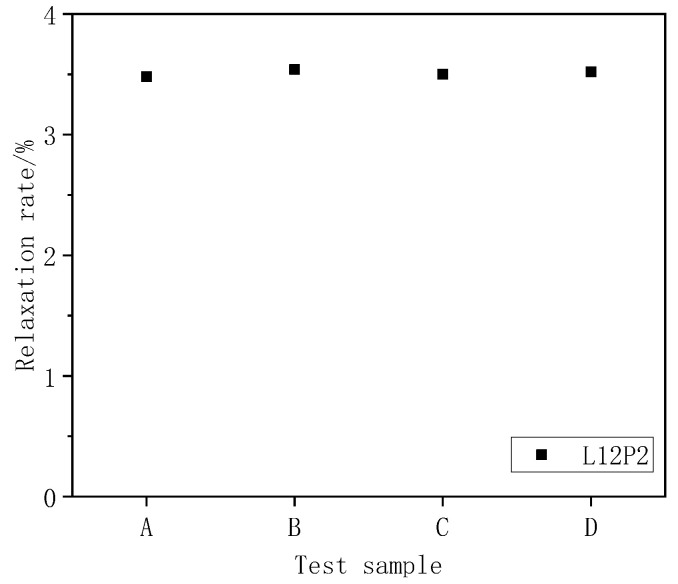
Test results of relaxation rate of L12P2 specimen.

To ensure the reliability of the test results, we pre-made four sets of L12P2 specimens and performed a 168 h relaxation test. The results demonstrate that the relaxation loss rate of the four sets of L12P2 specimens was almost the same (see Figure 3).

For coating CFRP cloth, first, the CFRP cloth was put into the tensioning anchor on the new tensioning table for pre-tensioning. Moreover, kraft paper was placed under the tensioning device to avoid the impregnation of the adhesive gluing to the new tensioning device. Then, the impregnating adhesive was applied to the CFRP evenly. Finally, after the impregnating adhesive was initially set, the tension was applied to the CFRP cloth.

### 2.2. Test Loading Method

**Figure 4:**
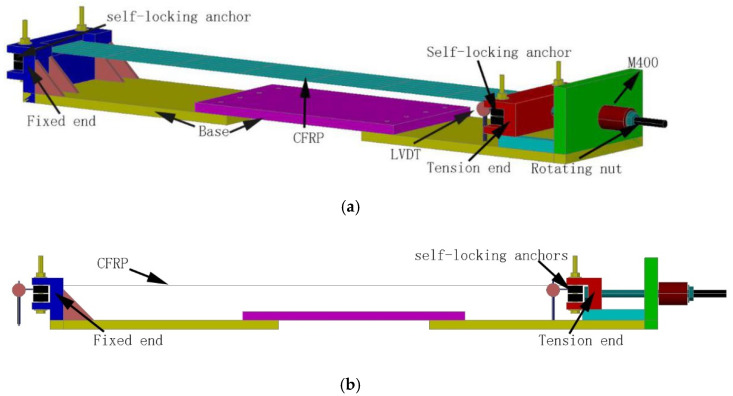
Prestressed CFRP cloth relaxation tensioning: (**a**) relaxation loss testing device of CFRP cloth; and (**b**) side view of the testing device.

**Figure 5:**
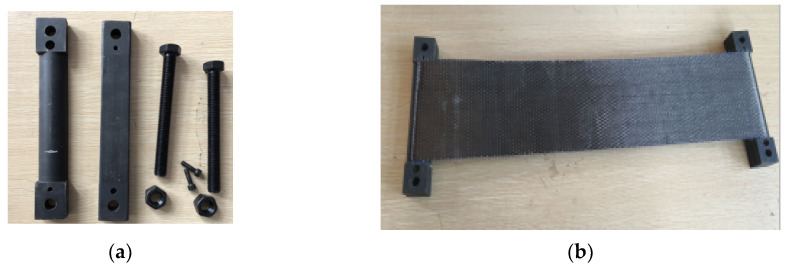
Picture of tension anchor: (**a**) anchorage; and (**b**) anchorage wrapped with a carbon fiber cloth.

The test was conducted in the Guangxi Key Laboratory of Rock and Soil Mechanics and Engineering, using a newly developed tensioning device [30], as shown in Figure 4a. The device consists of a fixed end (for fixing self-locking anchors), a tension end (used to apply prestress), a base, and an LVDT (Linear Variable Differential Transformer), where the position of the base can be adjusted according to the lengths of the test specimen. The two ends of the device are fixed to the CFRP by embedding self-locking anchors (Figure 5a). The self-locking anchor is fixed on the tensioning table by using high strength pins, and then one end is tensioned. Prestressing of the CFRP fabric is completed by tightening the nut at the tension end using a wrench. The amount of the slippage between the CFRP and the anchor end is measured by the LVDT existing at both ends of the tension device.

### 2.3. Test Measuring Method

**Figure 6:**
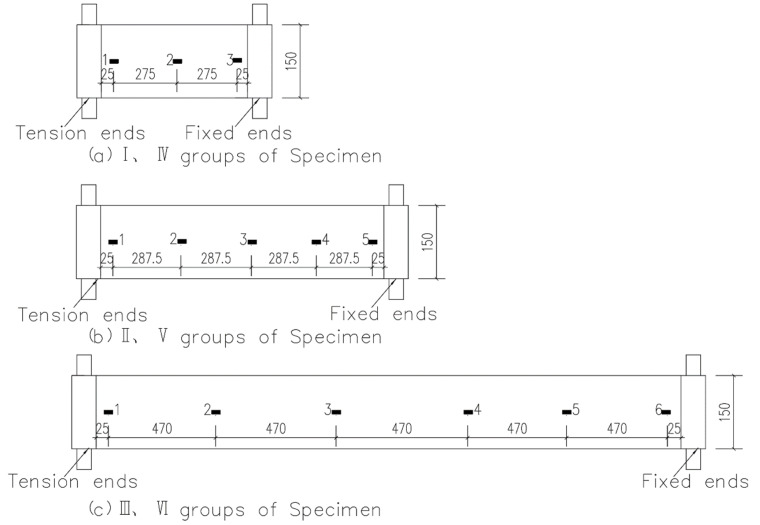
(**a**–**c**) Schematic of strain gauge arrangement on fiber (unit: mm).

The strain gauges in this test were numbered from “1” at the tension end. Three strain gauges were arranged on the CFRP cloth of Groups I and IV (Figure 6a); five strain gauges were arranged on the specimens of Groups II and V (Figure 6b); and six strain gauges were arranged on the specimens of Groups III and VI (Figure 6c). The strain gauges were pasted on the CFRP cloth by ethyl α-cyanoacrylate instant adhesive. The performance of CFRP cloth was not the same everywhere since CFRP cloth was a composite material woven from CFRP wire. To reduce this difference in performance, all strain gauges were pasted on the same group of CFRP wire. The strain gauges were connected to the DH3816 static resistance strain gauge Donghua Software Co., Ltd., Beijing, China. The control tension stress was measured by a force transducer and recorded by an M400 data acquisition system.

Before starting the test, the balance between the displacement sensor and the static strain collection system was first set to zero. The slippage between the CFRP and the anchor end was the value measured 1 h after the tension was completed. The data collection interval was set to 1 s, and then the wrench at the tension end was slowly rotated to apply prestress. When the prestress reached the set value, the prestress was no longer applied and the wrench was removed. In addition, the data collection interval was reset to 1 min. After data collection for 8 h, the collection interval was set to 1 h until the end of the test. Note that the whole test period was 168 h.

## 3. Test Results and Analysis

### 3.1. Stress Loss Analysis

In the axial tension test of CFRP cloth, the measured stress loss $\sigma_{R}$ divided into two parts: (1) the relaxation caused by the CFRP cloth itself, $\sigma_{1}$; and (2) the CFRP slip between the CFRP and the anchor end, $\sigma_{2}$

(1) $$\sigma_{R}=\sigma_{1}+\sigma_{2}$$

$\sigma_{2}$ caused by the slip between the CFRP and the anchor end is presented in Table 3. According to past domestic and foreign code for design [31,32], the slippage stress is calculated according to Equation (2).

(2) $$\sigma_{2}=E\frac{\Delta_{S}}{l}$$

The test results indicate that, when the CFRP cloth length and prestress level increased, the slippage between the CFRP and the anchor end increased. The glue coating had little impact on the slippage of CFRP cloth.

The relaxation rate of the CFRP cloth is defined as the ratio of the relaxation loss of CFRP cloth, $\sigma_{1}$, to the initial stress, $\sigma_{con}$ and the calculation equation is as follows:

(3) $$\beta =\frac{\sigma_{1}}{\sigma_{con}}$$

The test results are presented in Table 4.

### 3.2. Effect of Prestress Level on Relaxation Loss

**Figure 7:**
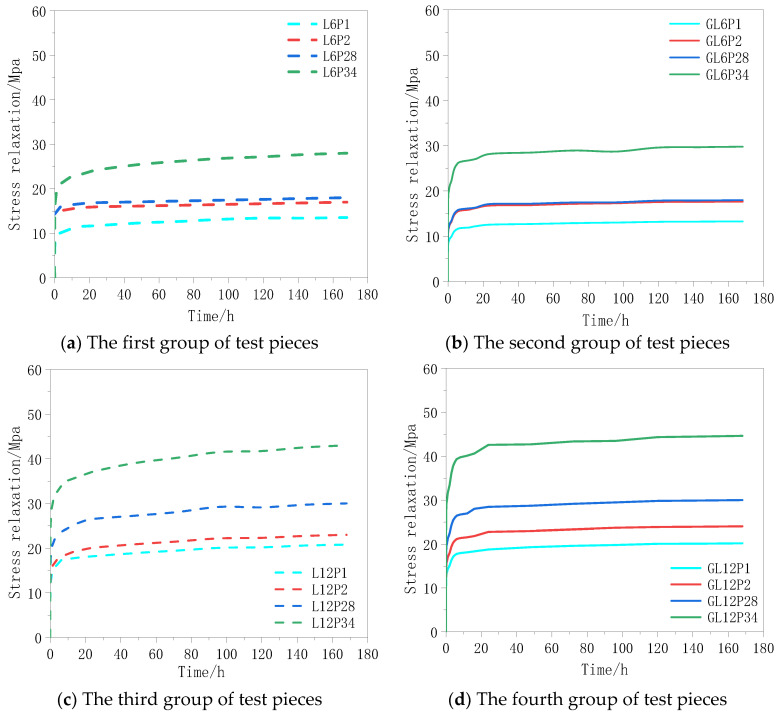

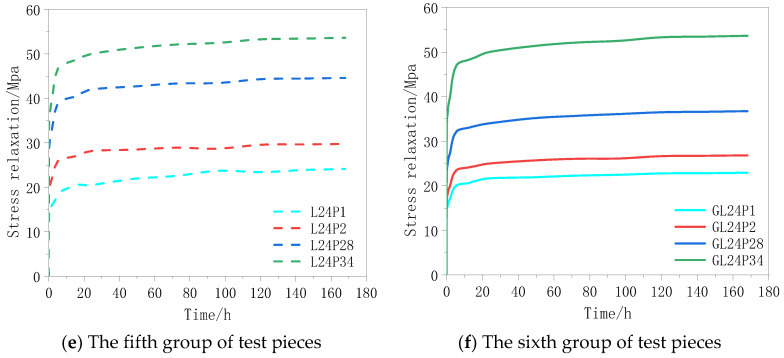
(**a**–**f**) Stress Relaxation loss-time relationship of CFRP cloth.

The relaxation curve of the test with the progress of time is shown in Figure 7. The analysis of Figure 7 and Table 4 suggests that:

(1) At the beginning of the test, the magnitude of stress relaxation of the CFRP fabric increased rapidly, and the relaxation rate raised promptly too. A shown in Figure 7a,c,e, the uncoated specimens could reach more than 85% of the final relaxation rate of the whole test period (168 h) just after 24 h. Figure 7b,d,f shows that, after 24 h, the glue-coated specimens could reach more than 95% of the final relaxation rate of 168 h. Thereafter, the relaxation rate gradually grew and ultimately tended to become stable.
(2) It can be found that, when the relaxation rate was increased from 0.1 to 0.3 fu, the prestress level of the test pieces of the three groups decreased significantly. When the prestress level was increased to 0.34 fu, the decrease in the relaxation rate was reduced substantially. We argue that this was because, when the prestress level was between 0.1 and 0.3 fu, as the prestress level increased, the curved fibers in the specimens became straightened, and the performance of the fiber cloth was improved. The larger is the prestress level, the more obvious this improvement will be. Hence, the relaxation rate raised with the prestress. When the prestress level was increased to 0.34 fu, some of the shorter fibers in the fiber cloth were broken resulting in the reduction of the performance of the fiber cloth. At this time, if the prestress level was raised further, the working performance of the fiber cloth not only did not increase but also decreased. Therefore, the relaxation rate of the specimen grew continuously with the increase of the prestress level.

### 3.3. Effect of Specimen Length on Relaxation Loss

**Figure 8:**
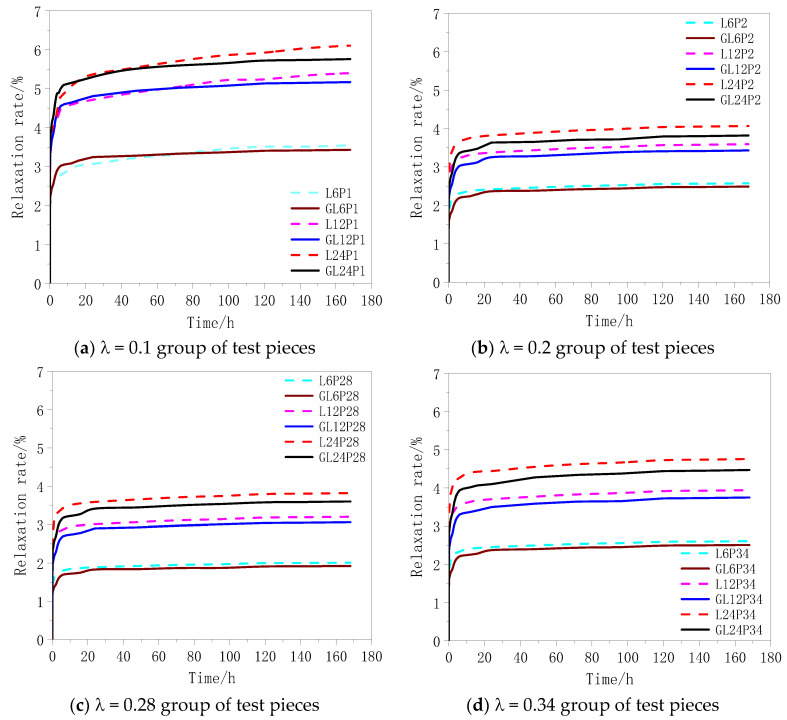
(**a**–**d**) Relaxation rate-time relationship of CFRP cloth.

The curves of the relaxation rate vs. time obtained from the tests are shown in Figure 8. The analysis of Figure 8 and Table 4 demonstrates that the relaxation rate became stable after 24 h. At the same prestress level, the relaxation rate increased as the CFRP fabric length increased. This was because some of the constituent filaments had some defects such as insufficient straightness and short length, originating from the manufacturing process. As the length of the CFRP fabric increased, the stress loss caused by the defect increased accordingly.

**Figure 9:**
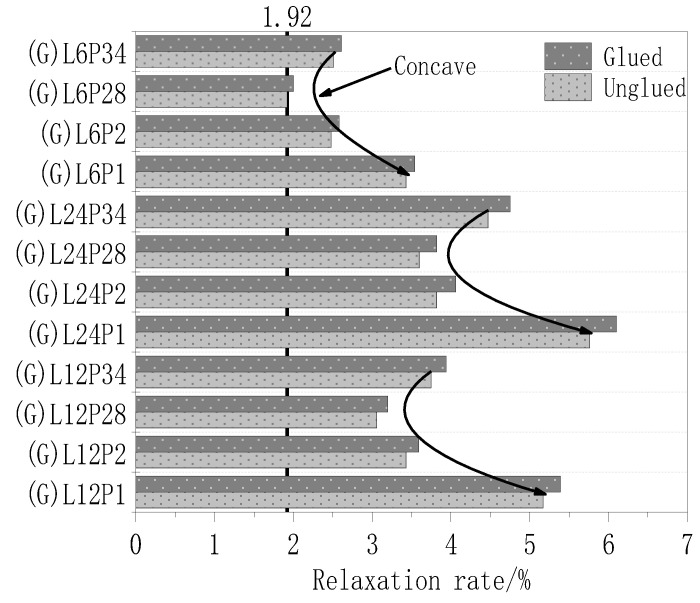
Post-168 h histogram of the relaxation rate of specimens under different treatments.

Figure 9 shows a histogram of the relaxation rate of the test pieces after 168 h under different treatment methods. Because the surface of the test piece was glue-coated, the fiber cloth was evenly stressed. Thus, the shorter fibers did not break due to uneven stress, and the overall performance was better. Under the same cloth length, the curve of relaxation rate vs. pre-stress level of the glue-coated test pieces was in the form of “concave”. With the rise of the prestress level from 0.28 to 0.34 fu, the relaxation rate of the test piece did not decrease but increased. Therefore, in engineering applications, when the prestress level is between 0.28 and 0.34 fu, coating the surface of the fiber cloth is useful for improving the performance of the fiber cloth.

### 3.4. Results Analysis of the Glue-Coated Test Pieces

Table 5 shows a comparison between the results of different processing methods. After gluing, the relaxation rate of the test piece was reduced by up to 6.28%.

### 3.5. Relaxation Rate-Time History at Different Positions of Carbon Fiber Cloth

**Figure 10:**
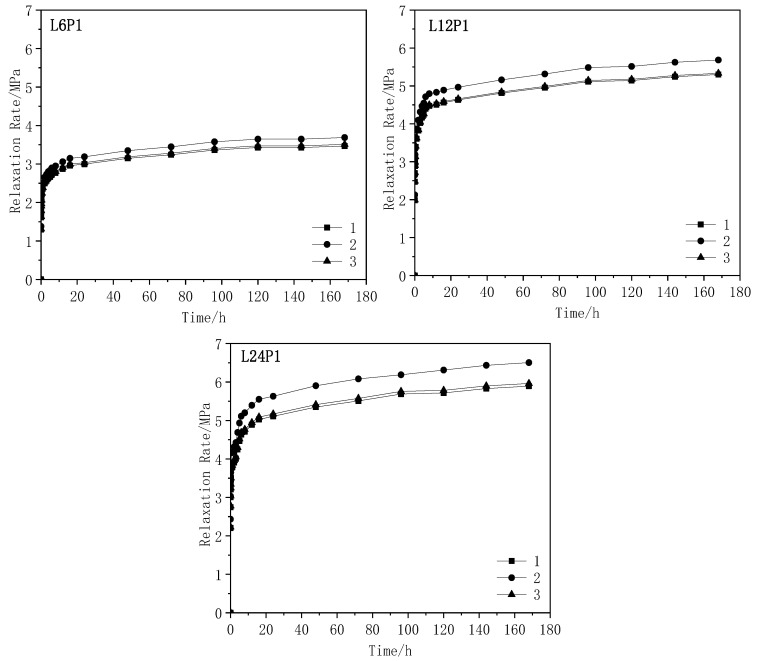
Time-dependent curves of relaxation rate at different locations of CFRP cloth.

Figure 10 exhibits the relationship between relaxation rate and time at different positions of the test pieces of each group. Here, only the control tension stress values at different positions of the horizontal test pieces under 0.1 fu prestressed level are shown in Table 6 (control tension stress equal to strain gauge reading at the corresponding position times elastic modulus of carbon fiber cloth). The control tension stress at the tension end and the fixed end of the specimen were large, and the difference between the two was not significant. The control tension stress in the middle of the cloth was smaller than that of the end of the test piece. The reason was that when the prestress was applied at the tension end, a reaction force equal to the value of the force applied at the tension end was also generated at the fixed end. As a result, the control tension stress at the fixed end, and the tension end were almost equal. Because some carbon fibers in the carbon fiber cloth were not straight enough, and there were gaps between the carbon fibers, these defects induced during the manufacturing process made a certain loss in the transmission of force on the carbon fibers. The closer it is to the middle of the cloth strip, the larger this loss would be. Therefore, the control tension stress in the middle of the cloth was smaller than the control tension stress at both ends. By reducing the loss in the middle of the CFRP cloth, the relaxation of the CFRP cloth could be effectively reduced and the utilization efficiency of the prestressed CFRP could be improved.

## 4. Calculation of Relaxation Rate

### 4.1. Shortcomings of Existing Relaxation Rate Equations

Li [28] proposed a equation for CFRP cloth relaxation rate changing with time through a 168 h experimental study on four CFRP cloth specimens:

(4) Y=a+blnt

where Y denotes the relaxation rate (%) and *t* stands for the time (h). *a* and *b* are statistically calculated based on the test results. The relaxation of CFRP fabrics develops fast in the early stage and tends to become gentle in the later stage. The coefficients *a* and *b* can be calculated through regression of the test results, and then the relaxation rate of CFRP fabrics in 10 and 50 years can be predicted accordingly. However, if the above equation is applied to calculate the relaxation rate of CFRP cloth, the coefficients *a* and *b* for each test piece are different, and, hence, its adaptability is poor.

In [25], based on the 2500 h experimental study on three CFRP cloth specimens, a CFRP cloth relaxation rate equation is proposed as follows:

(5) $$\beta =0.275\frac{f_{pi}}{f_{pu}}-0.083$$

where *β* is the relaxation rate (%) of CFRP cloth, $f_{pi}$ is the initial stress, and $f_{pu}$ is the ultimate tensile strength of the CFRP cloth. The prestress level has a significant influence on the relaxation rate of the CFRP cloth. However, the above equation only estimates the relaxation rate based on the prestress level, and the influential factors are poorly considered. Therefore, this relaxation rate equation is more in line with the aforementioned experimental results. However, it is not applicable to other experimental observations and, hence, its versatility is poor.

### 4.2. A New Relaxation Rate Equation

As mentioned above, the existing relaxation rate equations are generally not versatile due to weak consideration of the influencing factors. However, more consideration of the influencing factors makes the relaxation rate equation too complicated, which is not beneficial for guiding engineering practice. Based on the research results of Wu [33] combined with those obtained in this study (the relaxation rate of carbon fiber cloth with different prestress levels), the effect of the prestress level on the relaxation rate was clearly considered. Moreover, the influential factors of cloth length, time, and glue coating were introduced in the equation at the same time. The relaxation rates of carbon fiber cloth with different prestress levels are presented in Table 7.

Figure 9 shows that the relaxation rate of the test piece and the prestress level conform to a quadratic equation with one variable. Thus, it was assumed that the relationship of the relaxation rate with the prestress level, cloth length, time, and glue coating is as follows:

(6) $$\;\beta =g\delta \gamma \left(a\lambda^{2}+b\lambda +c\right)$$

where *λ* is the prestress level, *γ* is the influence coefficient of cloth length, *δ* is the influence coefficient of time, *g* is the influence coefficient of glue coating, and *a*, *b,* and *c* are undetermined constants.

**Figure 11:**
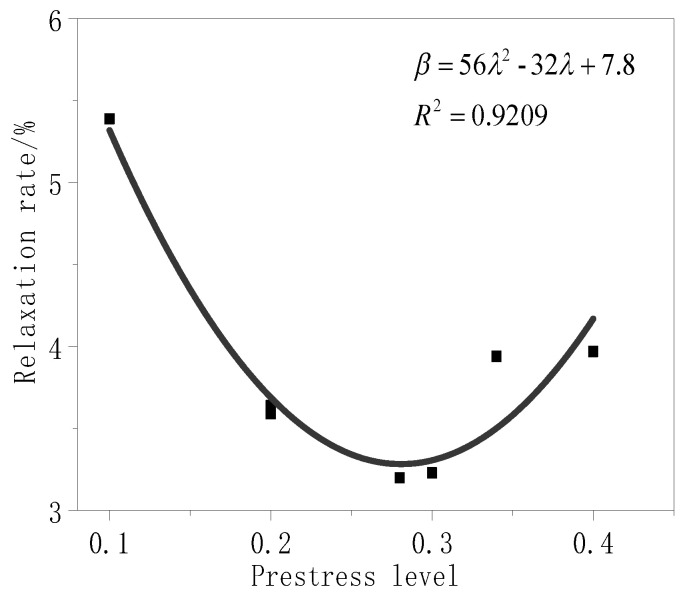
Relaxation curve.

Considering Equation (6), data analysis software (Origin, 2018C) was used to perform a non-linear regression analysis for the prestress level and relaxation rate data which are presented in Table 7 (refer to Figure 11). All undetermined constants could be obtained using this analysis (see Equation (7)). In the analysis, the fabric length *γ* of 1200 mm was taken as 1, *δ* was taken as 1 when the relaxation test time was 168 h, and *g* was taken as 1 when the surface of the test piece was untreated.

(7) $$\;\beta =\left(56\lambda^{2}-32\lambda +7.8\right)$$

With substituting the remaining testing results into Equation (6), the influence coefficient of the size on the relaxation rate after 168 h could be derived. When the specimen length was 600 and 2400 mm, *γ* was taken as 0.63 and 1.15, respectively.

Based on the test results of this research combined with the equation proposed in [28], the relaxation rate of each test piece at different test times could be calculated. When the test time was 2500 h, 10 years, and 50 years, *δ* was taken as 1.04, 1.21, and 1.30, respectively.

For the time being, there are few studies on the relaxation rate of CFRP cloth coated with glue. According to the test results of this research, the influence coefficient *g* of glue coating was taken as 0.962, 0.955, and 0.942 when the length of the test piece was 600, 1200, and 2400 mm, respectively.

## 5. Comparison of Calculated and Experimental Results

### 5.1. Comparison of the Calculated Values of the Relaxation Rate with Those of the Experimental Results 

To validate the relaxation rate calculation method proposed in this research, the calculated relaxation rates were compared with the experimental results obtained in this study, as indicated in Table 8. As is evident, the relaxation rates calculated by the proposed equation were in good agreement with the experimental results such that the degree of dispersion is minor. This suggests that the equation established in this research is appropriate for other similar tests as well $\beta_{e}/\beta_{c}$.

### 5.2. Comparison of Different Tests Results

Wang et al. [25] conducted a prestress loss test on reinforced concrete beams reinforced with four prestressed CFRP cloths. The fiber cloth length was 1200 mm, the cloth width was 140 mm, the thickness was 0.167 mm, the tensile strength was 3522 MPa, and the elastic modulus was 259 GPa. The prestress level was set to 0.28, 0.30, 0.36, and 0.4 fu, and the whole test time was 2500 h. This study only considered the stress loss results of three CFRP fabric test pieces after curing with epoxy resin for 72 h. For the test pieces that release prestress immediately after being stretched to the design prestress level, no comparison can be made here because of the small number of test pieces (one piece). In Table 9, it can be observed that the results of the relaxation rate equation proposed in this study were in good agreement with the test results in [24], with a maximum deviation of 1.11. This indicates that the proposed relaxation rate equation could accurately predict the relaxation loss of reinforced concrete beams reinforced by the post-tensioned CFRP cloth. 

Huang et al. [19] conducted 25 tests on reinforced concrete beams reinforced by prestressed CFRP slab. The specimens were divided into five groups, and each group had five specimens. Considering the effect of one-time over-tension and two-time over-tension on the test results, this research only compared the experimental and calculated results of one-time over-tension. The CFRP board was 1600 mm long, 100 mm wide, 0.23 mm thick, with a tensile strength of 3500 MPa, and an elastic modulus of 240 GPa. The prestress level was set to 0.2 and 0.3 fu, and the entire test time was 480 h. Since the CFRP board length was 1600 mm, the size influence coefficient *γ* was obtained by interpolation (*γ* = 1.05). By considering the calculated results of Table 9, it can be observed that the relaxation rates calculated by the proposed equation in this research had small deviations from the experimental results in [19]. The introduction of the size influence coefficient could even further reduce the discrepancies existing between experimental and calculated relaxation rates.

Li et al. [28] conducted four relaxation tests on prestressed CFRP fabrics. The tensile strength of CFRP fabrics was 3490 MPa and the elastic modulus was 220 GPa. The prestress level was set to 0.45 and 0.5 fu, and the total test time was 168 h. Table 9 suggests that, when the prestress level was 0.45 fu, the values of the relaxation rate predicted by the equation proposed in this research had a little deviation from the experimental results mentioned in [28]. When the prestress level was 0.5 fu, the deviation was larger. This was because, with the increase of the prestress level, the stress redistribution caused by the broken shorter fibers in the CFRP fabric was intensified, and, thus, the stress loss increased. Since the largest prestress level of the test piece in this study was 0.34, the derived relaxation rate equation could not accurately calculate the relaxation rate when the prestress level was 0.5 fu.

## 6. Conclusions

To understand the relaxation behavior of prestressed CFRP fabric under axial tension, six sets of stress relaxation tests were conducted in a constant temperature and humidity environment with a temperature of 20 ± 2 °C and relative humidity of 55–60%. Through the analysis of the experimental results, the following conclusions could be drawn:

(1) The relaxation rate of CFRP cloth was between 1.92% and 6.1%.
(2) After 24 h, the relaxation rate of CFRP cloth could reach more than 85% of the final relaxation rate of the whole test time (168 h). When the prestress level was less than 0.3 fu, the curved fibers in the CFRP cloth were straightened so that the performance of the CFRP cloth was fully enhanced and the relaxation rate decreased when the prestress level was increased. When the prestress level was greater than 0.3 fu, some fibers were broken. Thus, the stress redistribution occurred, and the relaxation rate increased as the prestress level was increased.
(3) As the size of CFRP fabric increased, defects such as insufficient straightness and short length caused during the fabrication process raised the stress loss. It was observed that, at the same prestress level, the relaxation rate decreased by a maximum of 47.6%.
(4) The relaxation loss was reduced by gluing of the test piece, and the relaxation rate was also diminished by 3.11–5.89% due to gluing. After the gluing treatment, the fiber cloth was evenly stressed, and the shorter fibers were not broken due to uneven stress. Hence, fiber cloth integrity was improved. In engineering practice, the size of the fiber cloth should be appropriately increased, and the pre-stress level should also be increased. In addition, the surface of the fiber cloth should be coated with glue to reduce the relaxation loss and to increase the effective prestress, which is advantageous for enhancing the reinforcement effect.
(5) According to the test results analysis, there were gaps between the sole carbon fabric after the prestress was applied. Moreover, there was a certain loss during the stress transmission process which made the CFRP cloth end stress greater and the middle stress smaller.
(6) The results of the equation established in this research can be used to predict the relaxation rate of CFRP cloth. These results were in good agreement with the experimental results. The deviation of the predicted results from the experimental results existing in the literature was small and the degree of dispersion was minor too. Therefore, the new equation proposed in this study can effectively predict the relaxation rate of the CFRP cloth.

## Figures and Tables

**Table 1:** Specimen parameters.

No.	Specimen No.	L/mm	λ	No.	Specimen No.	L/mm	λ
I	L6P1	600	0.1	IV	GL6P1	600	0.1
	L6P2	600	0.2		GL6P2	600	0.2
	L6P28	600	0.28		GL6P28	600	0.28
	L6P34	600	0.34		GL6P34	600	0.34
II	L12P1	1200	0.1	V	GL12P1	1200	0.1
	L12P2	1200	0.2		GL12P2	1200	0.2
	L12P28	1200	0.28		GL12P28	1200	0.28
	L12P34	1200	0.34		GL12P34	1200	0.34
III	L24P1	2400	0.1	VI	GL24P1	2400	0.1
	L24P2	2400	0.2		GL24P2	2400	0.2
	L24P28	2400	0.3		GL24P28	2400	0.3
	L24P34	2400	0.34		GL24P34	2400	0.34

Note: In GL6P1, G represents gluing treatment, L6 represents a tensile length of 600 mm, and P1 represents a prestress level of 0.1.

**Table 2:** Properties of materials.

Material Category	*f_y_*/MPa	*E*/MPa	*f_c_*/MPa
CFRP (sole fabric)	3634	2.38 × 10^5^	-
Dip adhesive	58	2584	78

Note: The CFRP model is CFS-I-300; CFS, carbon fiber sheet; I, High-intensity level 1; 300, Carbon fiber cloth weighs 300 g/m^3^.

**Table 3:** Slippage stress values.

Specimen No.	$\Delta_{s}/\mathrm{mm}$	$\sigma_{2}/\mathrm{MPa}$	Specimen No.	$\Delta_{s}/\mathrm{mm}$	$\sigma_{2}/\mathrm{MPa}$
L6P1	0.02	8.03	GL6P1	0.02	8.03
L6P2	0.03	12.05	GL6P2	0.03	12.05
L6P28	0.043	17.27	GL6P28	0.04	16.67
L6P34	0.06	24.1	GL6P34	0.055	22.09
L12P1	0.024	4.82	GL12P1	0.024	4.82
L12P2	0.033	6.62	GL12P2	0.033	6.62
L12P28	0.039	7.83	GL12P28	0.048	9.64
L12P34	0.052	10.44	GL12P34	0.046	9.24
L24P1	0.026	2.61	GL24P1	0.026	2.61
L24P2	0.038	3.81	GL24P2	0.035	3.51
L24P28	0.036	3.62	GL24P28	0.033	3.31
L24P34	0.058	5.82	GL24P34	0.052	5.22

**Table 4:** Stress relaxation loss β.

Specimen No.	$\sigma_{con}$ /MPa	$\sigma_{1-24}$ /MPa	$\frac{\sigma_{1-24}}{\sigma_{1-168}}$ /%	$\sigma_{1-100}/\mathrm{MPa}$	$\frac{\sigma_{1-100}}{\sigma_{1-168}}/\%$	$\sigma_{1-168}$ /MPa	$\sigma_{f}/\mathrm{MPa}$	β/%
L6P1	381.26	11.70	86.45	13.13	97.02	13.53	367.73	3.54
L6P2	690.28	15.39	86.45	17.02	95.58	17.80	672.20	2.58
L6P28	927.56	16.21	87.45	17.91	96.58	18.54	908.46	2.00
L6P34	1161.39	26.17	86.45	29.01	95.73	30.30	1130.70	2.61
L12P1	385.65	18.19	87.35	20.09	96.50	20.82	364.83	5.39
L12P2	670.87	21.01	87.35	23.23	96.58	24.05	645.95	3.59
L12P28	967.79	27.34	88.35	30.13	97.38	30.94	936.06	3.20
L12P34	1161.85	39.45	86.24	44.14	96.50	45.74	1115.26	3.94
L24P1	395.24	20.52	84.98	23.71	98.23	24.14	371.10	6.10
L24P2	669.89	23.20	85.43	26.68	98.24	27.16	641.84	4.06
L24P28	999.15	33.72	88.35	36.93	96.78	38.16	960.84	3.82
L24P34	1161.26	47.07	85.35	53.85	97.64	55.15	1105.85	4.75
GL6P1	386.32	12.53	94.59	12.99	98.06	13.25	373.07	3.43
GL6P2	708.67	16.76	95.10	17.26	97.90	17.62	690.37	2.48
GL6P28	935.76	17.09	95.20	17.47	97.36	17.95	917.04	1.92
GL6P34	1186.89	28.17	94.63	28.69	96.38	29.76	1156.23	2.51
GL12P1	396.48	18.77	93.00	19.79	98.06	20.19	376.29	5.17
GL12P2	700.00	22.79	94.90	23.69	98.66	24.01	675.99	3.43
GL12P28	980.90	28.43	94.82	29.45	98.23	29.98	950.01	3.06
GL12P34	1190.37	42.56	95.38	43.45	97.38	44.62	1145.37	3.75
GL24P1	397.65	21.67	94.60	22.46	98.06	22.90	374.75	5.76
GL24P2	702.00	25.53	95.20	26.11	97.36	26.82	675.18	3.82
GL24P28	1020.78	34.81	94.82	36.07	98.23	36.72	983.28	3.60
GL24P34	1200.23	51.32	95.68	52.50	97.88	53.64	1146.36	4.47

**Table 5:** Comparison of the relaxation rate of the unglue specimen (subscripted “u”) and the relaxation rate of the glue specimen (subscripted “G”).

Specimen No.	$\beta_{u}/\%$	Specimen No.	$\beta_{G}/\%$	$(\frac{\beta_{u}-\beta_{G}}{\beta_{G}})/\%$
L6P1	3.54	GL6P1	3.43	3.21
L6P2	2.58	GL6P2	2.48	4.03
L6P28	2.00	GL6P28	1.92	4.17
L6P34	2.61	GL6P34	2.51	3.98
L12P1	5.39	GL12P1	5.17	4.26
L12P2	3.59	GL12P2	3.43	4.67
L12P28	3.20	GL12P28	3.06	4.58
L12P34	3.94	GL12P34	3.75	5.07
L24P1	6.10	GL24P1	5.76	5.90
L24P2	4.06	GL24P2	3.82	6.28
L24P28	3.82	GL24P28	3.60	6.11
L24P34	4.75	GL24P34	4.47	6.26

**Table 6:** Control tension stress at different positions of the specimen (unit: MPa).

Strain Gauge No.	L6P1	L12P1	L24P1
1	390.42	392.83	409.7
2	367.04	-	-
3	385.6	366.32	371.14
5	-	390.42	
6	-	-	404.88

**Table 7:** The relaxation rate of carbon fiber cloth with different prestressing levels.

λ	Relaxation Rate of This Study β/%	Relaxation Rate of Existing Test Results β/%
0.1	5.39	-
0.2	3.59	3.64
0.28	3.20	-
0.3	-	3.23
0.34	3.94	-
0.4	-	3.97

Note: For all data shown in the table, cloth length was 1200 mm, test time was 168 h, and the test piece was unglued.

**Table 8:** Comparison of relaxation test values and calculation values.

Specimen No.	$\mathrm{Calculated}\;\mathrm{Relaxation}\;\mathrm{Rate}\;\beta_{e}/\%$	$\mathrm{Test}\;\mathrm{Relaxation}\;\mathrm{Rate}\;\beta_{c}/\%$	$\beta_{e}/\beta_{c}$	Specimen No.	$\mathrm{Calculated}\;\mathrm{RelaxationRate}\beta_{e}/\%$	$\mathrm{Test}\;\mathrm{Relaxation}\;\mathrm{Rate}\;\beta_{c}/\%$	$\beta_{e}/\beta_{c}$
L6P1	3.25	3.54	0.92	GL6P1	3.38	3.43	0.99
L6P2	2.58	2.24	1.15	GL6P2	2.38	2.48	0.96
L6P28	2	2.04	0.98	GL6P28	2.12	1.92	1.10
L6P34	2.61	2.48	1.05	GL6P34	2.22	2.51	0.89
L12P1	5.16	5.39	0.96	GL12P1	5.40	5.17	1.05
L12P2	3.59	3.64	0.99	GL12P2	3.81	3.43	1.11
L12P28	3.2	3.23	0.99	GL12P28	3.38	3.06	1.11
L12P34	3.94	3.56	1.11	GL12P34	3.55	3.75	0.95
L24P1	5.93	6.10	0.97	GL24P1	6.30	5.76	1.09
L24P2	4.06	4.19	0.97	GL24P2	4.44	3.82	1.16
L24P28	3.82	3.73	1.02	GL24P28	3.94	3.6	1.10
L24P34	4.75	4.24	1.12	GL24P34	4.14	4.47	0.93
AV			1.01	AV			1.03
SD			0.077	SD			0.094
COV			0.075	COV			0.091

**Table 9:** Comparison of experimental results in different literature.

Author	Specimen No.	λ	Time/h	$\mathrm{Documented}\;\mathrm{Relaxation}\;\mathrm{Rate}\;\beta_{c}/\%$	$\mathrm{Calculated}\;\mathrm{Relaxation}\;\mathrm{Rate}\;\;\beta_{e}/\%$	$\beta_{e}/\beta_{c}$
Wang [25]	BPC-30-1	0.36	2500	3.9	3.6	0.92
	BPC-40-1	0.40		3.6	4.0	1.11
	BPC-30-2	0.28		3.7	3.3	0.89
Huang [19]	1	0.20	480	4.25	3.82	0.90
	2	0.20		3.85		0.99
	3	0.2		4.21		0.91
	4	0.30		3.32	3.4	1.02
	5	0.30		3.41		1.00
Li [28]	1	0.45	168	5.364	4.741	0.88
	2	0.45		4.772		0.99
AV						0.96
SD						0.069
COV						0.072

## Nomenclature

AV	Average value
COV	Coefficient of variation
*E*	Modulus of elasticity of materials
$f_{y}$	Tensile strength of materials
$f_{c}$	compressive strength of materials
$f_{u}$	Ultimate strength of materials
L	Tensile length of specimen
SD	Standard deviation
λ	Prestress level of CFRP
$\Delta_{S}$	The slippage between the CFRP and the anchor end
$\sigma_{con}$	Control tension stress of the CFRP cloth
$\sigma_{f}$	Eliminate loss of stress caused by relaxation, effective stress, $\sigma_{f}=\sigma_{con}-\sigma_{1-168}$
$\sigma_{R}$	Measured stress loss of the CFRP cloth
$\sigma_{1}$	Stress of the relaxation loss of the CFRP cloth
$\sigma_{2}$	Stress loss of the CFRP cloth caused by the slippage between the CFRP and the anchor end $\sigma_{2}=E\frac{\Delta_{S}}{l}$
$\sigma_{1-24}$	Stress of the relaxation loss of the CFRP cloth in 24 h
$\sigma_{1-100}$	Stress of the relaxation loss of the CFRP cloth in 100 h
$\sigma_{1-168}$	Stress of the relaxation loss of the CFRP cloth in 168 h
β	Relaxation rate of the CFRP cloth
$\beta_{c}$	Test relaxation rate of the CFRP cloth
$\beta_{e}$	Calculate relaxation rate of the CFRP cloth
$\beta_{G}$	Relaxation rate of glue application specimen
$\beta_{u}$	Relaxation rate of unglue application specimen
